# Genome-Wide Identification of Kiwifruit *SGR* Family Members and Functional Characterization of SGR2 Protein for Chlorophyll Degradation

**DOI:** 10.3390/ijms24031993

**Published:** 2023-01-19

**Authors:** Juan Luo, Muhammad Abid, Yi Zhang, Xinxia Cai, Jing Tu, Puxin Gao, Zupeng Wang, Hongwen Huang

**Affiliations:** 1 Lushan Botanical Garden, Chinese Academy of Sciences, Jiujiang 332900, China; 2 College of Life Science, Nanchang University, Nanchang 330031, China; 3 Engineering Laboratory for Kiwifruit Industrial Technology, Chinese Academy of Sciences, Wuhan 430074, China

**Keywords:** *Actinidia chinensis*, *Actinidia eriantha*, STAY-GREEN, AcSGR2, chlorophyll degradation metabolism

## Abstract

The STAY-GREEN (SGR) proteins play an important role in chlorophyll (Chl) degradation and are closely related to plant photosynthesis. However, the availability of inadequate studies on SGR motivated us to conduct a comprehensive study on the identification and functional dissection of SGR superfamily members in kiwifruit. Here, we identified five *SGR* genes for each of the kiwifruit species [*Actinidia chinensis* (Ac) and *Actinidia eriantha* (Ae)]. The phylogenetic analysis showed that the kiwifruit *SGR* superfamily members were divided into two subfamilies the SGR subfamily and the SGRL subfamily. The results of transcriptome data and RT-qPCR showed that the expression of the kiwifruit *SGRs* was closely related to light and plant developmental stages (regulated by plant growth regulators), which were further supported by the presence of light and the plant hormone-responsive *cis*-regulatory element in the promoter region. The subcellular localization analysis of the AcSGR2 protein confirmed its localization in the chloroplast. The Fv/Fm, SPAD value, and Chl contents were decreased in overexpressed *AcSGR2*, but varied in different cultivars of *A*. *chinensis*. The sequence analysis showed significant differences within AcSGR2 proteins. Our findings provide valuable insights into the characteristics and evolutionary patterns of *SGR* genes in kiwifruit, and shall assist kiwifruit breeders to enhance cultivar development.

## 1. Introduction

Chlorophyll (Chl), a common photosynthetic pigment in plants, is indispensable for absorbing light energy and transferring electrons in the photosynthetic system [1]. The Chl metabolism at different developmental stages of plants is regulated by a series of complex molecular regulatory mechanisms [2]. During autumn, the color change from green to other colors in leaves (senescence) and fruits (fruit ripening) is directly related to Chl degradation [3,4]. The content of Chl in leaves also changes in the presence of biotic or abiotic stresses [5]. In higher plants, Chl is classified into Chl a and Chl b [6]. Chl a exists in both the central complexes and the light-harvesting complexes, while Chl b only exists in the light-harvesting complexes [7]. Chl is a potential cytotoxic and free Chl, and its derivatives must be removed immediately [2]. Therefore, degradation of Chl is essential under certain conditions and it is generally carried out by the pheophorbide a oxygenase (PAO)/phyllobilin pathway [2]. The first step in the PAO pathway is catalyzed by magnesium dechelatase [an enzyme encoded by Mendel’s green cotyledon gene also called STAY-GREEN (SGR)] to remove magnesium (Mg^2+^) from Chl a [8].

The SGR superfamily is divided into two subfamilies, including the SGR subfamily and the SGR-like subfamily (SGRL). So far, *SGR* genes have only been identified in a few plants, including three *SGR* genes in *Arabidopsis thaliana* [8], three genes in *Solanum Lycopersicum* [9], and four genes in *Cucumis melo* [10]. The SGR gene family members have a great influence on the physiological functions of plants. In tomato and pepper, a non-synonymous mutation of Arg to Ser, at amino acid residues 143 of SGR protein, directly led to the maintenance of the green flesh of tomato and retention of Chl in pepper [11]. In soybean, the deletion of 42 amino acid residues in SGR protein resulted in the development of a new variety possessing a stay-green trait, which enhanced photosynthetic efficiency and yield [12]. In tomatoes, the overexpression of *SlSGRL*, which was regulated by *SlABI5/SlABI5-LIKE* (ABA-INSENSITIVE5), promoted ABA-mediated Chl degradation more than in wild type plants by interacting with *SlPPH* (pheophytin pheophorbide hydrolase). In addition, *SlLHCa2* (light-harvesting complex a2) was involved in ABA-induced Chl degradation [13]. In pakchoi, the early termination of the *SGR* gene (normal SGR had 265 amino acids and mutant SGR had 113 amino acids) resulted in the stay-green trait, which delayed post-harvest yellowing and extended the shelf life of vegetables [14,15]. In cucumber, a non-synonymous mutation of Glu to Arg at amino acid residue 108 lost the expression sensitivity of CsSGR protein, and improved the disease resistance of cucumber [16]. The knockout null lines (SlSGR1-KO) of tomato accumulated significantly higher Chl and had more numbers of differentially expressed genes (DEGs) related to photosynthesis and chloroplast than WT tomato plants [17]. In *Arabidopsis thaliana*, BALANCE of CHLOROPHYLL METABOLISM1 (BCM1) and BCM2 regulated the activity of SGR to control the content of chlorophyll in leaves [18]. The overexpression of *CsSGR* in *A. thaliana* and *N. bethamiana* indicated its involvement in degradation of photosynthetic Chl complexes [19].

Kiwifruit (commonly known as Chinese gooseberry) is an important commercial fruit plant. In the past few decades, the world’s kiwifruit industry has witnessed a boom in production [20]. There are 54 species and about 70 taxa in *Actinidia* which vary greatly in fruit size, shape, fruit color, nutritional value, and shelf life [21]. Our current study mainly focused on the regulatory mechanism of Chl degradation metabolism in fruits. Previously, the *SGR2* had a higher expression at the fruit de-greening stage in *A. chinensis* (a yellow-fleshed kiwifruit), suggesting that *SGR* is an important regulator in kiwifruit Chl degradation [22]. However, the characteristics and regulatory role of the *SGR* gene family members for Chl degradation in kiwifruit are unclear.

Therefore, we conducted a genome-wide identification of the *SGR* gene family members in red-fleshed and green-fleshed cultivars from *A. chinensis* and *A. eriantha* with contrast Chl retention during fruit ripening, respectively [23]. We analyzed the gene structure, protein characteristics, evolutionary relationships, and gene expression profiles in different tissues and under different treatments to elucidate the structural and functional evolution of the *SGR* gene family in kiwifruit. The regulatory role of *SGR2* genes from two different varieties of *A*. *chinensis* was investigated by transient overexpression in tobacco leaves. Our study provides valuable insights into the potential role of *SGR2* in regulating Chl metabolism in kiwifruit. In addition, the benchmark data provided in the present study shall assist kiwifruit breeders to improve existing germplasm resources.

## 2. Results

### 2.1. Genome-Wide Identification and Phylogenetic Analysis of Kiwifruit SGRs

**Figure 1:**
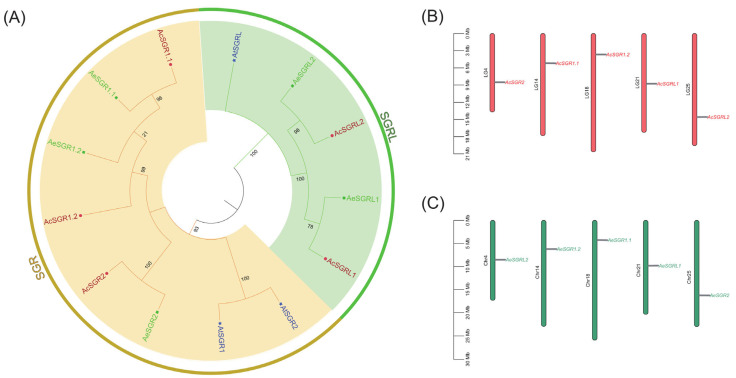
The analysis of SGR superfamily members in kiwifruit. (**A**) Phylogenetic relationships of SGR protein sequences, (**B**) chromosomal distribution of *SGR* genes in *A. chinensis* genome, and (**C**) chromosomal distribution of *SGR* genes in *A. eriantha* genome. The SGR protein sequences from *Arabidopsis thaliana* (At) were represented by blue color, those in *A. chinensis* (Ac) were represented by deep red color, and those in *A. eriantha* (Ae) were represented by green color. The SGR and SGRL subfamily were highlighted in grey and green colors, respectively.

We identified five potential SGR proteins in each kiwifruit species (Ac and Ae) by blasting query AtSGR protein sequences in the kiwifruit genome database (KGD) (Table S1). The candidate kiwifruit SGR proteins were named after the homologous proteins in *Arabidopsis thaliana*. Protein sequences of AcSGRs, AeSGRs, and AtSGRs were used for phylogenetic analysis to explore the evolutionary relationship of the kiwifruit SGRs. Based on phylogenetic analysis, the kiwifruit SGR proteins were divided into two distinct subfamilies, namely SGR and SGRL (Figure 1A). The results revealed that both *A. chinensis* and *A. eriantha* had three members in the SGR subfamily, and in contrast, only two members in the SGRL subfamily. The *SGR* genes were equally distributed on five chromosomes, but one gene was on each chromosome of each species. Interestingly, both species have the same five chromosome 4, 14, 18, 21, and 25 possessing the *SGR* gene (Figure 1B).

### 2.2. Gene Structure Analysis and Conserved Domain Distribution of SGR in Kiwifruit

**Figure 2:**
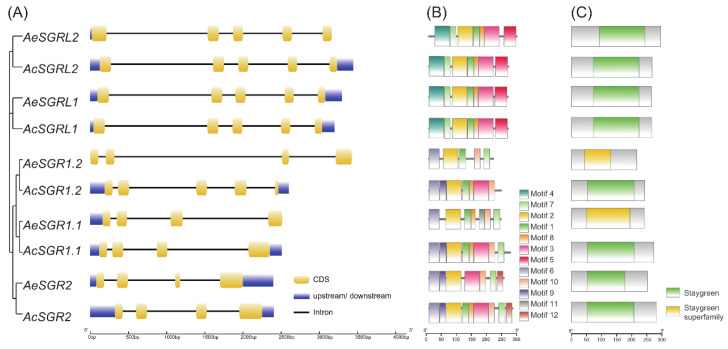
Gene structure and conserved motif architecture of SGR family members in two kiwifruit species. (**A**) the exon-intron structure, (**B**) the conserved motifs distribution, and (**C**) the presence of the conserved domain.

The intron-exon structure analysis of *SGR* genes revealed that all members from SGR subfamily had three introns and four exons, except *AcSGR1.2* which had four introns and five exons. However, in the SGRL subfamily, all members had four introns and five exons (Figure 2A). The length of introns and exons varied greatly within kiwifruit *SGRs* (Figure 2A), suggesting that structural changes in gene sequences might have functional differences.

We further analyzed the conserved motifs of kiwifruit SGR protein to explore their structural diversity and functional differentiation. A total of 12 different conserved motifs (motifs 1–12) were identified in kiwifruit SGRs, and motif logos were presented in the supplementary data (Figure S1). The motif 2 was located inside the conserved domains (staygreen or staygreen superfamily) of all kiwifruit SGRs (Figure 2B,C). The distribution of motifs was similar within a subfamily and vice versa. In both kiwifruit species, the number of motifs in SGRL was the same for each member, but the number of motifs was different in SGR subfamily members (Figure S2).

### 2.3. Analyses of Cis-Acting Regulatory Elements in the Promoter Region

**Figure 3:**
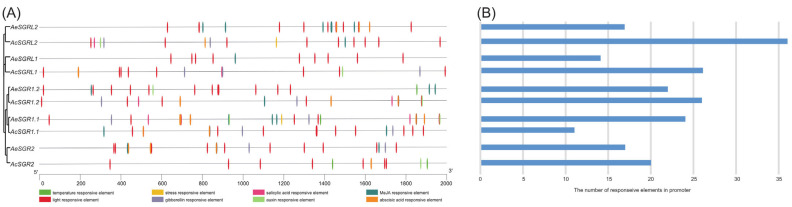
Analysis of *cis*-acting regulatory elements in the promoter regions of kiwifruit *SGR*s. (**A**) The distribution of *cis*-elements in the 2000 bp upstream promoter regions of *SGR* genes. Different *cis*-elements are represented by different colors, and (**B**) the number of responsive *cis*-elements in each *SGR* gene promoter.

We identified 119 and 94 *cis*-acting regulatory the elements in the 2000 bp upstream sequence of *AcSGRs* and *AeSGRs*, respectively. The *cis*-acting regulatory elements were temperature-responsive, stress-responsive, salicylic acid-responsive, MeJA-responsive, light-responsive, gibberellin-responsive, auxin-responsive, and abscisic acid-responsive. The light-responsive elements were the most commonly occurring, indicating that light significantly regulated the expression of the kiwifruit *SGRs* (Figure 3A). In Ac, *AcSGRL2* contained the highest number of *cis*-acting elements (36 elements), but *AcSGR1.1* contained the lowest number (11 elements) (Figure 3B). Similarly, in Ae, *AeSGR1.1* contained the highest number of *cis*-acting elements (24 elements) and *AeSGRL1* contained the lowest number (14 elements) (Figure 3B). Furthermore, the distribution of the *cis*-acting elements varied greatly within an ortholog gene pair (i.e., *AcSGR2* and *AeSGR2*) (Figure 3A).

### 2.4. Synteny Analysis for Kiwifruit SGRs

**Figure 4:**
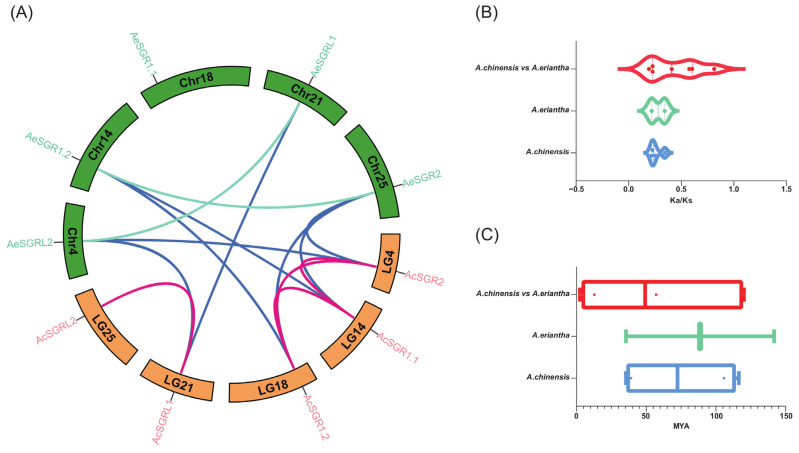
Collinearity analysis of kiwifruit *SGRs*. (**A**) The syntenic gene pairs were connected by lines with different colors. The orange and green bars indicated chromosomes for Ac and Ae, respectively. Red lines represented the synteny relationship in Ac; Green lines represented the synteny relationship in Ae; Blue lines represented syntenic gene pairs in Ac and Ae. (**B**) Ka/Ks ratio, and (**C**) divergence time of syntenic gene pairs.

The collinearity analysis was performed to identify duplicated gene pairs for *SGR* genes within (Ac and Ae) and between (Ac vs. Ae) two kiwifruit species. There were four homolog gene pairs in Ac, two homolog gene pairs in Ae, and seven ortholog gene pairs between Ac and Ae (Figure 4). The Ka/Ks ratio of the duplicated gene pairs ranged from 0.19 to 0.81, indicating that these gene pairs underwent purifying selection (Table S2). The results also suggested that the duplication events in the kiwifruit genome occurred about 4.09 to 141.78 million years ago (MYA), and the genome duplication events occurred earlier in Ae (141 MYA) than in Ac (116 MYA) (Table S2).

### 2.5. Differences in AcSGR Genes Expression under Biotic and Abiotic Stresses

**Figure 5:**
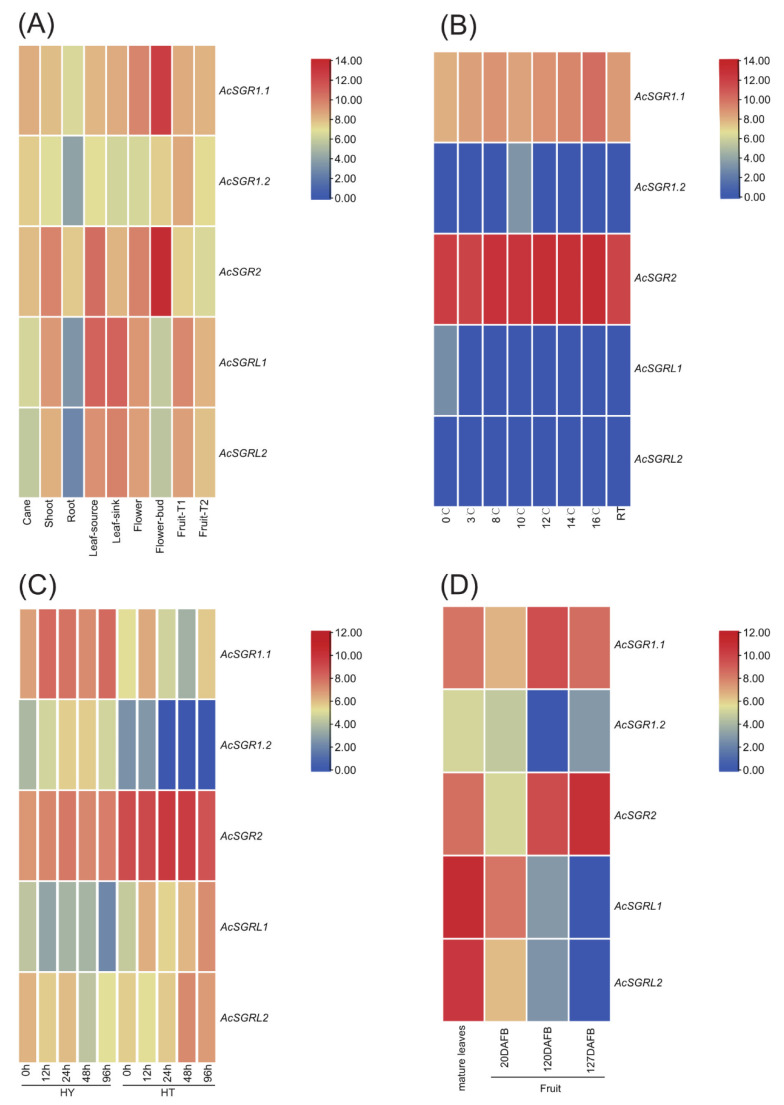
Expression profiles of *AcSGR* family members. (**A**) In different plant tissues from *A. chinensis* (T1: no ethylene production, T2: autocatalytic ethylene production). (**B**) In mature fruit of ‘Hort16a’ exposed to eight different temperature intervals in storage for two days. °C, degrees celsius; RT, room temperature. (**C**) In shoot of a susceptible cultivar (Hongyang, HY) and a resistant (Huate, HT) cultivar infected with *Psa*. The h represents the hours. (**D**) In fully expanded leaves and different fruit developmental stages of ‘Hongyang’. DAFB, days after the full bloom of fruit. The bar at the right of each heat map represents expression values.

We used four different transcriptome datasets to analyze the differences in the expression profile of five *AcSGR* in eight different tissues (cane, shoot, root, leaf-source, leaf-sink, flower, flower bud, and fruit under different developmental stages). The results showed that the expression of *AcSGRs* was highly specific to different tissues and fruit developmental stages. The *AcSGRs* were expressed less in the root than in other tissues. Notably, the expression of *AcSGR2* was lower in the T1 and T2 than in other developmental stages of fruit (Figure 5A). In comparison to the low temperatures that reduced the content of Chl [24], we explored *AcSGRL1* and *AcSGR1.2* which had higher expressions at 0 °C and 10 °C, respectively (Figure 5B). The amount of Chl contents significantly affecting plant resistance to diseases had been reported [25]. We then evaluated the expression changes of *AcSGRs* in a *Psa.* resistant cultivar (Haute, HT), and a susceptible cultivar (Hongyang, HY) after inoculation with *Pseudomonas syringae* pv. actinidiae (*Psa.*). The results revealed that the expression level of the *AcSGRL1* and *AcSGRL2* genes in HT was significantly up-regulated after *Psa.* inoculation. Moreover, the expression levels of *AcSGR1.1* and *AcSGR1.2* were lower in HT than in HY (Figure 5C), suggesting that these genes may be chosen as candidate genes for breeding disease-resistant kiwifruit varieties in the future. We further profiled *AcSGRs* in leaves (green color), fruits at the immature stage (green color), and at the mature stage (not green color). The results showed that the expression patterns of *AcSGR1.2*, *AcSGRL1,* and *AcSGRL2* were different in the leaves and in the mature stage of fruits. The expression of *AcSGRL1* and *AcSGRL2* genes in fruits was quite opposite to that of *AcSGR2*. In contrast, the expression patterns of *AcSGR1.1* and *AcSGR2* remained the same in leaves and in the mature stage of fruits (Figure 5D).

### 2.6. RT-qPCR Analysis of Kiwifruit SGR Genes

**Figure 6:**
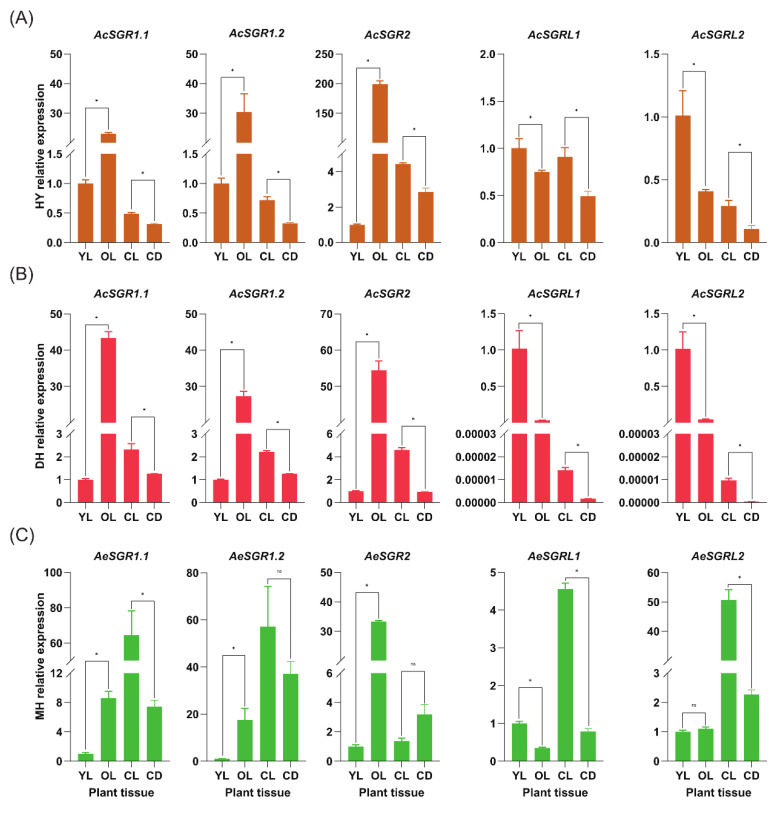
Expression profile analysis of *AcSGR* and *AeSGR* genes by using RT-qPCR in different plant tissues. Expression profile of *SGRs* (**A**) for HY, (**B**) for DH, and (**C**) for MH. The results were presented as the mean ± SD of three replicates. *Actin* was used as the internal standard for each gene, and the 2^−ΔΔCt^ method was used to calculate the relative expression of candidate genes. “*” indicated significant differences at *p* < 0.05, and “ns” indicated non-significant differences at *p* > 0.05. OL, old leaves; YL, young leaves; CD, callus tissues under dark condition; CL, callus tissues under light condition; DH, ‘Donghong’; HY, ‘Hongyang’; MH, ‘Maohua no.1’.

We carried out RT-qPCR analysis for five *AcSGR* genes and five *AeSGR* genes in old leaves (OL), young leaves (YL), callus under light conditions (CL), and callus under dark conditions (CD). The samples used were taken from two varieties of *A. chinensis* (DH ‘Donghong’, and HY ‘Hongyang’) and one variety of *A. eriantha* (MH ‘Maohua no.1′) (Figure S3). The expression of SGR subfamily members was revealed as up-regulated, and for SGRL subfamily members was down-regulated with the aging of leaves, except for *AeSGRL2* (Figure 6 and Figure S6). The expression of all kiwifruit *SGR* genes in CD was significantly lower than that in CL, excluding *AeSGR1.2* and *AeSGR2*. In Ac, most of the *SGRs* showed higher expression in leaves than in calluses (Figure 6A,B). In contrast, all the *AeSGRs* had higher expression in calluses than leaves, except for *AeSGR2* (Figure 6C), indicating that the expression of *SGR*s was species-specific. It was worth noting that the kiwifruit *SGR2* had the greatest effect on the Chl degradation in leaves, so we chose this gene for the subsequent experiments.

### 2.7. Subcellular Localization and Transient Overexpression of AcSGR2 in Tobacco Leaves

**Figure 7:**
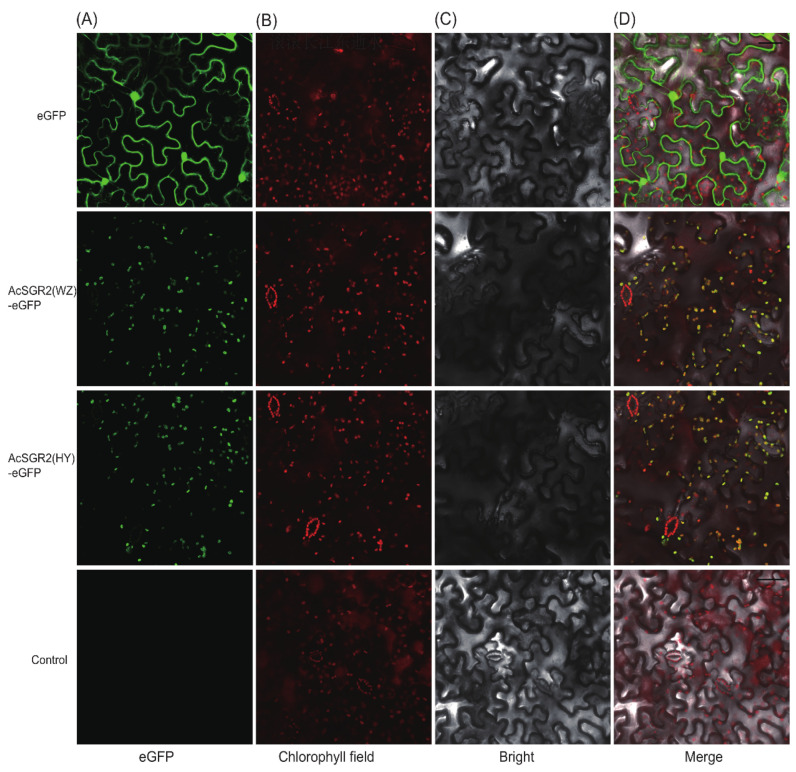
Subcellular localization of the fusion protein 35S:: AcSGR2-eGFP in tobacco leaves. Images were taken under (**A**) fluorescence, (**B**) Chl field, (**C**) bright field, and (**D**) Merged field. The Chl autofluorescence was used to localize chloroplasts. WZ, ‘Wuzhi no.3’; HY, ‘Hongyang’; eGFP, enhanced green fluorescence protein; AcSGR2(WZ), AcSGR2 from WZ; AcSGR2(HY), AcSGR2 from HY; Bars = 50 μm.

**Figure 8:**
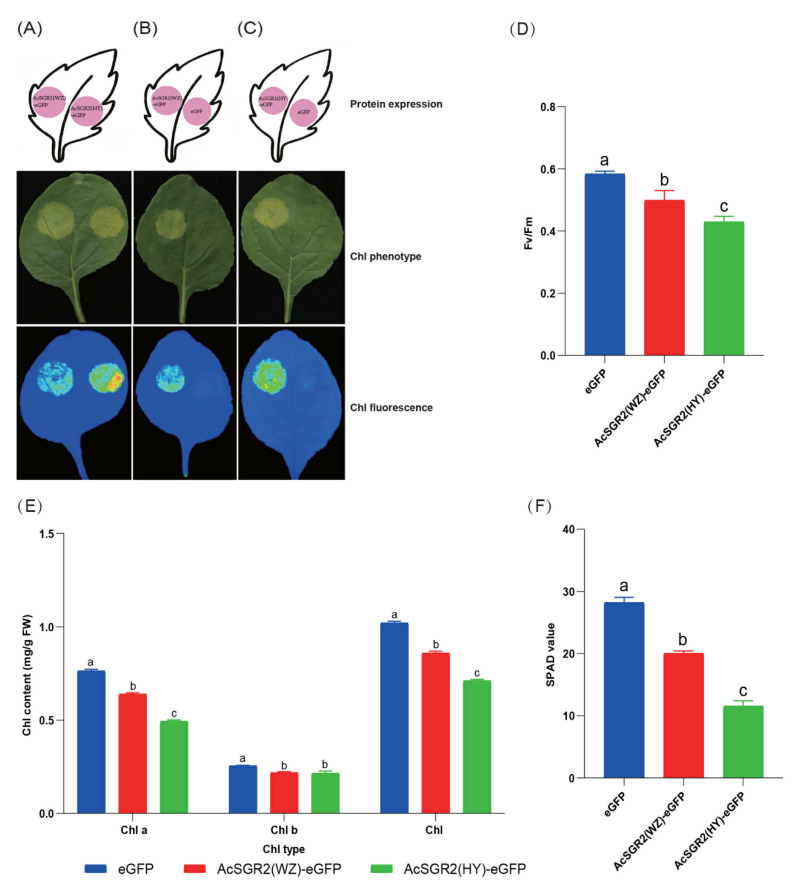
Transient overexpression of *AcSGR2* in tobacco leaves. Detection of Chl degradation in 6-weeks-old soil-grown tobacco leaves (**A**) overexpressing 35S::AcSGR2(WZ)-eGFP and 35S::AcSGR2(HY)-eGFP, (**B**) overexpressing 35S::AcSGR2(WZ)-eGFP and 35S:: eGFP, and (**C**) overexpressing 35S::AcSGR2(HY)-eGFP and 35S:: eGFP. (**D**) Fv/Fm ratio. (**E**) Chl content, and (**F**) SPAD value. WZ, ‘Wuzhi no.3’; HY, ‘Hongyang’; eGFP, enhanced green fluorescence protein; AcSGR2(WZ), AcSGR2 from WZ; AcSGR2(HY), AcSGR2 from HY. The results were presented as the means ± SD of three replicates. The mean differences were identified with different letters based on the results of Tukey’s multiple range test (*p* < 0.05).

The experiment was conducted by using two cultivars of *A. chinensis*, HY (Hong yang, a red-fleshed cultivar), and WZ (Wuzhi no.3, a green-fleshed cultivar) to clone the coding sequences (CDS) of the *AcSGR2* gene. On basis of the CDS, we identified four SNPs (single nucleotide polymorphism) between HY and WZ, and there were two amino acids (aa) non-synonymous mutations at 101aa (Leu “L” to Val “V”) and 108 aa (Arg “R” to Lys “K”) (Figure S4) in protein sequences of AcSGR2. In silico subcellular localization analysis of the AcSGR2 protein predicted its presence in the chloroplast. We further verified the localization of the AcSGR2 protein by using sub-cellular localization analysis in tobacco leaves (Figure 7). The transient overexpression of the *AcSGR2* from HY and WZ were performed in tobacco leaves, revealing that the AcSGR2 protein from HY significantly degraded Chl in tobacco leaves compared to AcSGR2 from WZ (Figure 8 and Figure S5). Moreover, AcSGR2 from HY significantly decreased Fv/Fm, Chl contents, and SPAD value in tobacco leaves than AcSGR2 from WZ. We speculated that the aa change at two positions (both were present inside the staygreen conserved domain, 101aa was inside motif 2, and 108aa was inside motif 1) might be responsible for the functional differentiation (Figure 2C).

## 3. Discussion

The Chl degradation, a multi-step catabolic pathway, plays an important role in leaf senescence and fruit ripening, and it is regulated by SGRs [26]. The biological function of SGR proteins is well documented for chlorophyll degradation in leaves (during senescence) and fruit (during ripening) [27]. The loss of functional or functionally defective SGR proteins led to the stay-green trait or delay in Chl degradation in leaves [28,29]. Similarly, the loss of SGRs function in the tomato led to brown colored fruit by promoting carotenoid accumulation and delaying Chl degradation [30]. In the current study, the overexpression of the *AcSGR2* gene promoted Chl degradation in tobacco leaves, suggesting that *AcSGR2* gene is key gene for Chl degradation in kiwifruit. Therefore, genetic manipulation of the *AcSGR2* gene may regulate Chl degradation and affect kiwifruit productivity.

The Chl synthesis pathway is well understood, but the Chl degradation pathway is less studied and warrants further research [31]. Recently, the discovery of Chl Mg^2+^-dechelatase was a revolutionary event that greatly facilitated our understanding of the Chl degradation pathway [8]. Chl Mg^2+^-dechelatase is a member of the *SGR* gene superfamily which has been extensively studied in many plant species [32]. Here, we identified the *SGR* superfamily members in two different kiwifruit species, which had a higher number of *SGR* genes than that in *Arabidopsis thaliana*. We speculated that the results might be a part of evolutionary history because kiwifruit had experienced three whole genome duplications (WGDs), while *Arabidopsis thaliana* had only two WGD [33]. In addition, the Ka/Ks ratios in both kiwifruit species were < 1 which suggest that the *SGR* genes underwent purifying selection in the kiwifruit SGRs, when the conserved nature of kiwifruit SGR family members eliminated the chances of occurrence of non-synonymous mutations in them [34,35].

Due to the indispensable role of Chl in photosynthesis, Chl non-degradation mutants have been found in many crop plants, but some of these mutants are caused by the mutation of *SGRs* [36]. Obviously, the Chl non-degradation mutants obtained advantages in yield, disease resistance, and environmental tolerance, proving Chl non-degradation traits to be of great importance for plant breeding [32,37,38]. Genus *Actinidia*, including kiwifruit, has a great diversity of genetic resources with Chl degradable and non-degradable fruit types, contrasting with normal Chl degradation of kiwifruit leaves [39]. This phenomenon offers a great opportunity to explore transcription and post-transcription differentiation in Chl degradation to improve the stay-green trait and the production of kiwifruit. For this purpose, we have used two different cultivars including HY (red-fleshed fruit) and WZ (green-fleshed fruit) to dissect the regulatory molecular mechanism of the *SGR2* gene underlying Chl degradation. The occurrence of SNPs in the kiwifruit genome is a common phenomenon because of the vast wild-type genetic resources and the presence of different polyploidy levels [40]. The SNPs in DNA sequences occasionally cause a change in protein sequence, which impart different tolerance abilities in plants to adverse environmental factors [41]. The aa change in the AcSGR2 protein sequence might alter the tertiary structures, thereby causing functional diversity [42]. We obtained similar results in the current study where *AcSGR2* from HY degraded a greater amount of Chl than that of WZ, suggesting that the functional studies of *AcSGR2* genes from different germplasm resources of kiwifruit can improve our understanding about Chl degradation.

The binding sites composition for pre-transcriptional regulation was entirely different for *SGRs* from Ac and Ae, suggesting the diverse responses of *SGRs* from different species to different stimuli. The hormone-responsive elements in the promoter region assisted the plants in sensing the emergency signals and triggering the transcription of *SGRs*. Additionally, the environment-responsive factors in the promoter region helped plants to regulate *SGRs* expression to adapt to adverse environmental conditions. Previous studies have found that the ethylene pathway, abscisic acid pathway, and light signaling pathway intersected to regulate Chl degradation in a changing environment [43,44]. Therefore, we hypothesized that the regulatory role of *SGRs* for Chl degradation in kiwifruit depends on both environmental factors and phytohormones. Our findings by RT-qPCR confirmed the hypothesis on the regulation of kiwifruit *SGRs* under diverse environmental conditions. For an instance, the relative expression of most *AcSGRs* was constantly low in CD due to a lack of light signals for light-responsive elements in the promoter region and a lack of Chl. Similar results for Chl metabolism were reported in fruits of kiwifruit under bagging treatment (dark conditions) [45]. We believe that the promoter region of *AcSGR2* must also have many changes between HY and WZ which may prove to be an important source of information for future research on the transcriptional regulation of *AcSGR2*.

Since the SGR subfamily is extensively studied compared to the SGRL subfamily, it will be worthwhile to decipher the regulatory role of SGRL in Chl metabolism [32,46]. We observed significant changes in the relative expression of SGRL subfamily members in different plant tissues under diverse environmental conditions. We believe that the functional studies for SGRL subfamily members will open up new horizons for the regulation of Chl metabolism in plants [47]. Additionally, it will be interesting to know whether SGR subfamily and SGRL subfamily members (containing same conserved domain) have a complementary effect on their role for Chl degradation in kiwifruit under certain circumstances.

## 4. Materials and Methods

### 4.1. Identification of SGR Family Members in the Kiwifruit Genome

All the sequences for kiwifruit and *A. thaliana* were retrieved from Kiwifruit Genome Database (KGD, http://kiwifruitgenome.org/, accessed on 10 January 2023) and The Arabidopsis Information Resources (TAIR, https://www.arabidopsis.org/, accessed on 10 January 2023), respectively. The SGR protein sequences from *A. thaliana* were used as a query by the local BLASTp tool with a cutoff score of ≥100 and an e-value of ≤1× 10^−10^ to construct the SGR proteins database for both kiwifruit species. The candidate *AcSGRs* and *AeSGRs* were identified by determining the conserved domain for kiwifruit SGR via the Conserved Domain Database (CDD, https://www.ncbi.nlm.nih.gov/Structure/cdd/cdd.shtml, accessed on 10 January 2023) and the Simple Modular Architecture Research Tool (SMART, http://smart.embl.de/, accessed on 10 January 2023). The protein sequences containing the Staygreen domain or Staygreen superfamily domain were retained for subsequent analysis.

### 4.2. Protein Structure Analysis for Kiwifruit SGR

We utilized the ExPASY server (http://web.expasy.org/protparam/, accessed on 10 January 2023) to compute the sequence length, theoretical isoelectric point (pI), grand average of hydropathicity (GRAVY), and molecular weight (MW) of the kiwifruit SGR proteins.

### 4.3. Analysis of Kiwifruit SGRs Structure, Motif features, and Cis-Elements in the Promoter Region

The genome sequences and coding sequences of the kiwifruit *SGR* genes were used to investigate gene structures (intron-exon composition) in kiwifruit *SGRs* by using the Gene Structure Display Server (GSDS 2.0, http://gsds.cbi.pku.edu.cn/, accessed on 10 January 2023). We identified a maximum of 12 conserved motifs for kiwifruit SGR proteins by using MEME suit (http://meme-suite.org/tools/meme, accessed on 10 January 2023) [48]. The PlantCARE database [49] was utilized to predict *cis*-regulatory elements in the 2000 bp upstream promoter region of kiwifruit *SGR* genes.

### 4.4. Phylogenetic Analysis of Kiwifruit SGRs

The multiple sequence alignments for SGR proteins from kiwifruit and *A. thaliana* were performed by using ClustalX software with default parameters [50]. The MEGA X software was utilized to construct a phylogenetic tree by using the neighbor-joining (NJ) method with 1000 bootstrap replicates.

### 4.5. Chromosomal Location, Gene Duplication, and Synteny Analysis for Kiwifruit SGRs

We obtained the location of kiwifruit *SGR* genes on the chromosome from the GFF file by using an in-house Perl script, and the genes were visualized on chromosomes by using MapGene2 Chrome software (http://mg2c.iask.in/mg2c_v2.0/, accessed on 10 January 2023). The duplication patterns for *SGRs* were identified by producing syntenic blocks within and between kiwifruit genomes by using MCScan software with default parameters [51], and duplicated gene pairs of kiwifruit *SGRs* were visualized by using TBtools [52]. The calculations for substitution rates in duplicated *SGR* gene pairs were performed by using TBtools software [52].

### 4.6. Expression Analysis of Kiwifruit SGRs

We retrieved four published transcriptome datasets (PRJNA691387, PRJNA514344, PRJNA187369, and PRJNA514180) from NCBI (https://www.ncbi.nlm.nih.gov/, accessed on 10 January 2023) to investigate the expression profiles of kiwifruit *SGRs*. All the transcriptome datasets were re-analyzed by using *Actinidia chinensis*, ‘Red5’ as a reference genome [33,53]. The raw reads were aligned by using the HISAT2 v2.0.1 [54], and the transcripts were assembled and quantified by using the STRINGTIE v2.1.5 [55].

### 4.7. Collection of Plant Samples

The samples from fresh leaves of *A. eriantha* ‘Maohua no.1’ and *A. chinensis* ‘Hongyang’, ‘Donghong’, and ‘Wuzhi no.3’ were obtained from Kiwifruit Germplasm Resources Nursery of Lushan Botanical Garden, Nanchang County, Jiangxi Province, China. Samples were divided into two parts to clone genes and induct calluses [56]. The kiwifruit calluses were treated with light and dark conditions and samples were used for subsequent study [57]. The tobacco plants were grown in a growth chamber under the following conditions: 16/8-h-light/dark photoperiod, 250 μmmol photons m^−2^ s^−1^, 26 °C, and 55% humidity.

### 4.8. RNA Extraction and cDNA Synthesis

Total RNA was extracted with a Hipure Plant RNA Mini Kit (Magen, Shanghai, China). Similarly, the cDNA was synthesized by using a Trans Script One-Step gDNA Removal and cDNA Synthesis Super Kit (Transgen, Beijing, China).

### 4.9. Quantitative Real-Time PCR (RT-qPCR) Analysis

The specific pair of primers for kiwifruit *SGR* genes were designed by Bioedit software and cDNA from kiwifruit leaves and callus was used as a template to perform RT-qPCR analysis (Table S3). The reaction mixture for RT-qPCR was prepared according to the manufacturer’s instructions of Perfect Start Green qPCR SuperMix (Transgen, Beijing, China). The kiwifruit actin gene was used as an internal control to calculate the ΔCt values of target genes. The 2^−ΔΔCt^ method was used to calculate the relative expression of candidate genes and the specificity of the amplification was determined by observing the melting curve [58].

### 4.10. Subcellular Localization of AcSGR2

The coding sequences (CDS) of the *AcSGR2* lacking stop codon from HY and WZ were ligated into the pGreen vector to construct the 35S:: AcSGR2(HY)-eGFP and 35S:: AcSGR2(WZ)-eGFP fusion protein. The 35S:: eGFP (an empty vector) was used as a negative control. The subcellular localization analysis was performed by introducing the constructed vector into young tobacco leaves by *Agrobacterium tumefaciens*-mediated transformation [59]. The eGFP protein signals were detected under a laser scanning confocal microscope (Olympus, Tokyo, Japan) as described previously [60,61].

### 4.11. Transient Over-Expression Analysis of AcSGR2 in Tobacco Leaves

The vectors from subcellular localization analysis were reused to perform the transient overexpression analysis. The experiment was performed in 6-week-old tobacco plants by selecting healthy leaves at the fourth internode (counting from the shoot tip) for bacterial culture inoculation. Chl contents, Fv/Fm, and SPAD value in transformed tobacco leaves were determined according to previously described methods [62].

### 4.12. Statistical Analysis

All experimental data were analyzed with GraphPad Prism v9 software. Mean differences were compared by using Tukey’s tests [63], and the differences were considered statistically significant at *p* < 0.05.

## 5. Conclusions

SGR proteins are key enzymes in Chl degradation metabolism. In the current study, we comprehensively analyzed the evolutionary classification, conserved domain, gene structure, and expression characteristics of the kiwifruit *SGR* superfamily members. We have reported the key function of *AcSGR2* for chlorophyll degradation in tobacco leaves; however, it will be interesting to know the function of *AcSGR2* in kiwifruit, particularly in fruit coloration. Additionally, it will be of great importance to obtain *AcSGR2* knockout plants to develop the functional stay-green trait in kiwifruit. Our work provided valuable information for the evolutionary patterns and functional differentiation of *SGRs*, and we have proposed a set of candidate genes for kiwifruit breeding programs in the future.

## Data Availability

All the data used in this article is available at Kiwifruit Genome Database (KGD) and The Arabidopsis Information Resources (Tair) online databases.

## Supplementary Materials

The following supporting information can be downloaded at: https://www.mdpi.com/article/10.3390/ijms24031993/s1.
