# Supplementary material for: Genome-Wide Identification of Kiwifruit SGR Family Members and Functional Characterization of SGR2 Protein for Chlorophyll Degradation
Source: Int J Mol Sci. 2023 Jan 19;24(3):1993. doi: 10.3390/ijms24031993 (PMC9917040; doi:10.3390/ijms24031993)
Supplement: Supplementary file 1 [file ijms-24-01993-s001.zip › Figure S4.pdf]

(A)

```
Reference      ATGGGTACTCTGACCCCTCCTCTCGTGCTTCCATCAGTGTTCAAGCGTCTCTCTGTCTCTAAACAACATCATAAAAGTCC   80
AcSGR2(HY)     ATGGGTACTCTGACCCCTCCTCTCGTGCTTCCATCAGTGTTCAAGCGTCTCTCTCTCTCTAAACAACATCATAAAAGTCC   80
AcSGR2(WZ)     ATGGGTACTCTGACCCCTCCTCTCGTGCTTCCATCAGTGTTCAAGCGTCTCTCTCTCTCTAAACAACATCATAAAAGTCC   80
Consensus      atgggtactctgacccctcctctcgtgcttccatcagtgttcaagcgtctctct tctctaaacaacatcataaaagtcc

Reference      TCTCTTCATCTACAGAAGAAGCAAGAAGCATCAATCTTTAATCCCTGTTGCTCGATTGTTTGGGCCAGCAATATTTGAAG  160
AcSGR2(HY)     TCTCTTCATCTACAGAAGAAGCAAGAAGCATCAATCTTTAATCCCTGTTGCTCGATTGTTTGGGCCAGCAATATTTGAAG  160
AcSGR2(WZ)     TCTCTTCATCTACAGAAGAAGCAAGAAGCATCAATCTTTAATCCCTGTTGCTCGATTGTTTGGGCCAGCAATATTTGAAG  160
Consensus      tctcttcatctacagaagaagcaagaagcatcaatctttaatccctgttgctcgattgtttgggccagcaatatttgaag

Reference      CATCGAAGCTTAAGGTTTTGTTTCTTGGAATTGATGAGAAGAACCATCCAGAGAAGCTTCCAAGAACTTACACACTCACA  240
AcSGR2(HY)     CATCGAAGCTTAAGGTTTTGTTTCTTGGAATTGATGAGAAGAACCATCCAGAGAAGCTTCCAAGAACTTACACACTCACA  240
AcSGR2(WZ)     CATCGAAGCTTAAGGTTTTGTTTCTTGGAATTGATGAGAAGAACCATCCAGAGAAGCTTCCAAGAACTTACACACTCACA  240
Consensus      catcgaagcttaaggttttgtttcttggaattgatgagaagaaccatccagagaagcttccaagaacttacacactcaca

Reference      CACAGTGATGTGACCTCTAAAATCACTCTGGCCATCTCTCAAACCATTAACAATTCCCAGTTACAGGGTTGGTACAATAA  320
AcSGR2(HY)     CACAGTGATGTGACCTCTAAGATCACTCTGGCCATCTCTCAAACCATTAACAATTCCCAGTTACAGGGTTGGTACAATAA  320
AcSGR2(WZ)     CACAGTGATGTGACCTCTAAAATCACTCTGGCCATCTCTCAAACCATTAACAATTCCCAGGTACAGGGTTGGTACAATAA  320
Consensus      cacagtgatgtgacctctaa atcactctggccatctctcaaaccattaacaattcccag tacagggttggtacaataa

Reference      GTTCCAGAGAGATGAAGTGGTTGCAGAGTGGAGGAAGGTGAAGGGGAAAATGTCTCTCCATGTTCATTGTCACATAAGTG  400
AcSGR2(HY)     GTTCCAGAGAGATGAAGTGGTTGCAGAGTGGAGGAAGGTGAAGGGGAAAATGTCTCTCCATGTTCATTGTCACATAAGTG  400
AcSGR2(WZ)     GTTCCAGAGAGATGAAGTGGTTGCAGAGTGGAAGAAGGTGAAGGGGAAAATGTCTCTCCATGTTCATTGTCACATAAGTG  400
Consensus      gttccagagagatgaagtggttgcagagtgga gaaggtgaaggggaaaatgtctctccatgttcattgtcacataagtg

Reference      GTGGTCACTTCTTATTGGATTTGTGTGCTAGGCTCAGATACTTCATCTTCTACAAAGAACTCCCAGTGGTTTTGAAGGCC  480
AcSGR2(HY)     GTGGTCACTTCTTATTGGATTTGTGTGCTAGGCTCAGATACTTCATCTTCTACAAAGAACTCCCAGTGGTTTTGAAGGCC  480
AcSGR2(WZ)     GTGGTCACTTCTTATTGGATTTGTGTGCTAGGCTCAGATACTTCATCTTCTACAAAGAACTCCCAGTGGTTTTGAAGGCC  480
Consensus      gtggtcacttcttattggatttgtgtgctaggctcagatacttcatcttctacaaagaactcccagtggttttgaaggcc

Reference      TTTGTTCATGGAGATGGTGACTTGTTCAACAACTACCCAGAACTAGAGGAAGCCATGGTGTGGGTTTATTTCCACTCCAA  560
AcSGR2(HY)     TTTGTTCATGGAGATGGTGACTTGTTCAACAACTACCCAGAACTAGAGGAAGCCATGGTGTGGGTTTACTTCCACTCCAA  560
AcSGR2(WZ)     TTTGTTCATGGAGATGGTGACTTGTTCAACAACTACCCAGAACTAGAGGAAGCCATGGTGTGGGTTTACTTCCACTCCAA  560
Consensus      tttgttcatggagatggtgacttgttcaacaactacccagaactagaggaagccatggtgtgggttta ttccactccaa

Reference      CATACAAGAATTCAACAAGGTGGAGTGCTGGGGCCCGCTCAAGGAAGCTGCAGCACCTTCTACAGGGGGGATCAGTGGGG  640
AcSGR2(HY)     CATACAAGAATTCAACAAGGTGGAGTGCTGGGGCCCGCTCAAGGAAGCTGCAGCACCTTCTACAAGGGGGATCAGTGGGG  640
AcSGR2(WZ)     CATACAAGAATTCAACAAGGTGGAGTGCTGGGGCCCACTCAAGGAAGCTGCAGCACCTTCTACAAGGGGGATCAGTGGGG  640
Consensus      catacaagaattcaacaaggtggagtgctggggccc ctcaaggaagctgcagcaccttctaca gggggatcagtgggg

Reference      CCTACAAGGACAAAAGGAAGGAAGAACCAACAAGCGACTGTGAGATGCCACTGCCCTGCCAAGAGGACTGCACATGCTGC  720
AcSGR2(HY)     CCTACAAGGACAAAAGGAAGGAAGAACCAACAAGCGACTGTGAGATGCCACTGCCCTGCCAAGAGGACTGCACATGCTGC  720
AcSGR2(WZ)     CCTACAAGGACAAAAGGAAGGAAGAACCAACAAGCGACTGTGAGATGCCACTGCCCTGCCAAGAGGACTGCACATGCTGC  720
Consensus      cctacaaggacaaaaggaaggaagaaccaacaagcgactgtgagatgccactgccctgccaagaggactgcacatgctgc

Reference      TTTCCCCCAATGAGCATCATCCCATGGCCCCAGGAAGAGCCTCTTGGGACCCACCAAAACCAAACCCTAAACCAGCCAAA  800
AcSGR2(HY)     TTTCCCCCAATGAGCATCATCCCATGGCCCCAGGAAGAGCCTCTTGGGACCCACCAAAACCAAACCCTAAACCAGCCAAA  800
AcSGR2(WZ)     TTTCCCCCAATGAGCATCATCCCATGGCCCCAGGAAGAGCCTCTTGGGACCCACCAAAACCAAACCCTAAACCAGCCAAA  800
Consensus      tttcccccaatgagcatcatcccatggccccaggaagagcctcttgggacccaccaaaaccaaaccctaaaccagccaaa

Reference      CTACTTGGGAAGATCTGGTGGGACACTTTG                                                   830
AcSGR2(HY)     CTACTTGGGAAGATCTGGTGGGACACTTTG                                                   830
AcSGR2(WZ)     CTACTTGGGAAGATCTGGTGGGACACTTTG                                                   830
Consensus      ctacttgggaagatctggtgggacactttg
```

(B)

```
Reference      1  MGTLTPPLVLPSVFKRLSVSKQHHKSPLFIYRRSKKHQSLIPVARLFGPAIFEASKLKVLFLGIDEKNHPEKLPRTYTLT  80
AcSGR2 (HY)    1  MGTLTPPLVLPSVFKRLSLSKQHHKSPLFIYRRSKKHQSLIPVARLFGPAIFEASKLKVLFLGIDEKNHPEKLPRTYTLT  80
AcSGR2 (WZ)    1  MGTLTPPLVLPSVFKRLSLSKQHHKSPLFIYRRSKKHQSLIPVARLFGPAIFEASKLKVLFLGIDEKNHPEKLPRTYTLT  80
                                    19

Reference     81  HSDVTSKITLAISQTINNSQLQGWYNKFQRDEVVAEWRKVKGKMSLHVHCHISGGHFLLDLCARLRYFIFYKELPVVLKA  160
AcSGR2 (HY)   81  HSDVTSKITLAISQTINNSQLQGWYNKFQRDEVVAEWRKVKGKMSLHVHCHISGGHFLLDLCARLRYFIFYKELPVVLKA  160
AcSGR2 (WZ)   81  HSDVTSKITLAISQTINNSQVQGWYNKFQRDEVVAEWKKVKGKMSLHVHCHISGGHFLLDLCARLRYFIFYKELPVVLKA  160
                                      101            108

Reference    161  FVHGDGDLFNNYPELEEAMVWVYFHSNIQEFNKVECWGPLKEAAAPSTGGISGAYKDKRKEEPTSDCEMPLPCQEDCTCC  240
AcSGR2 (HY)  161  FVHGDGDLFNNYPELEEAMVWVYFHSNIQEFNKVECWGPLKEAAAPSTRGISGAYKDKRKEEPTSDCEMPLPCQEDCTCC  240
AcSGR2 (WZ)  161  FVHGDGDLFNNYPELEEAMVWVYFHSNIQEFNKVECWGPLKEAAAPSTRGISGAYKDKRKEEPTSDCEMPLPCQEDCTCC  240
                                                                209

Reference    241  FPPMSIIPWPQEEPLGTHQNQTLNQPNYLGRSGGTL*  277
AcSGR2 (HY)  241  FPPMSIIPWPQEEPLGTHQNQTLNQPNYLGRSGGTL*  277
AcSGR2 (WZ)  241  FPPMSIIPWPQEEPLGTHQNQTLNQPNYLGRSGGTL*  277
```
