# Supplementary material for: Genome-Wide Identification of Kiwifruit SGR Family Members and Functional Characterization of SGR2 Protein for Chlorophyll Degradation
Source: Int J Mol Sci. 2023 Jan 19;24(3):1993. doi: 10.3390/ijms24031993 (PMC9917040; doi:10.3390/ijms24031993)
Supplement: Supplementary file 1 [file ijms-24-01993-s001.zip › Supplementary captions.pdf]

Figure S1. Sequence logos for 12 conserved motifs identified in the kiwifruit SGR proteins.
Figure S2. Motif number in each subfamily (A) for Ac and (B) for Ae.
Figure S3. Photos of different tissues of kiwifruit for RT-qPCR. OL, old leaves; YL, young leaves; CD, callus tissues under dark condition; CL, callus tissues under light condition; DH, 'Donghong'; HY, 'Hongyang'; MH, 'Maohua no.1'. Scale bar for capturing photos was set to 50 μm.
Figure S4. AcSGR2 sequence alignment from HY and WZ. (A) nucleotide sequence alignment; (B) amino acid sequence alignment. The reference sequence was Red5, Sequence was the same in HY and WZ but different from Red5, expressed in green color; Sequence was different in HY and WZ expressed in red color.
Figure S5. Transient expression of AcSGR2(WZ) and AcSGR2(HY) in tobacco leaves. (A), (B) and (C) represented three biological replicates.
Figure S6. Expression analysis of *AcSGR* and *AeSGR* genes using RT-qPCR in different tissues. (A) and (D) for HY, (B) and (E) for DH, (C) and (F) for MH. The results were presented as the mean ± SD of three replicates. Actin was used as the internal standard for each gene. * indicated significant differences at $p < 0.05$, ** $p < 0.01$, *** $p < 0.001$, **** $p < 0.0001$. OL, old leaves; YL, young leaves; CD, callus tissues under dark condition; CL, callus tissues under light condition; DH, 'Donghong'; HY, 'Hongyang'; MH, 'Maohua no.1'.
Table S1. Protein composition and physicochemical characteristics of kiwifruit SGRs.
Table S2. The syntenic gene pairs identified in kiwifruit *SGRs*.
Table S3. All the primers were used in this study.
