# Supplementary figures and images for: Genome-Wide Identification of Kiwifruit SGR Family Members and Functional Characterization of SGR2 Protein for Chlorophyll Degradation
Source: Int J Mol Sci. 2023 Jan 19;24(3):1993. doi: 10.3390/ijms24031993 (PMC9917040; doi:10.3390/ijms24031993)

Motif 1

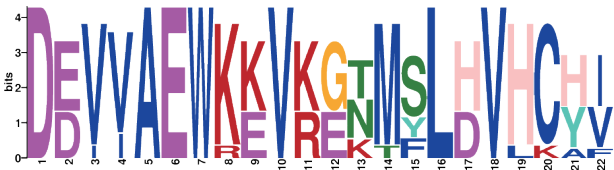

Motif 2

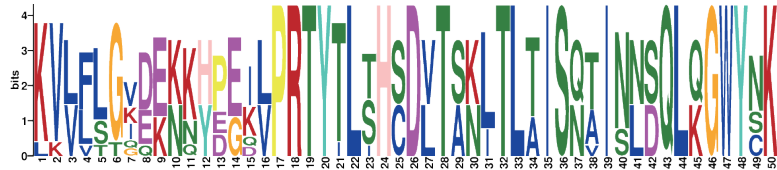

Motif 3

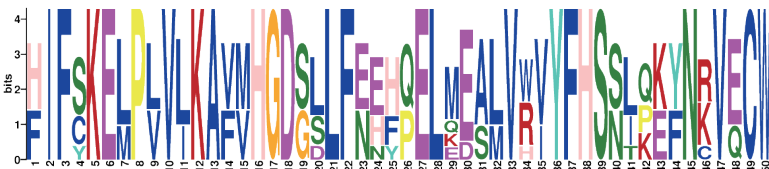

Motif 4

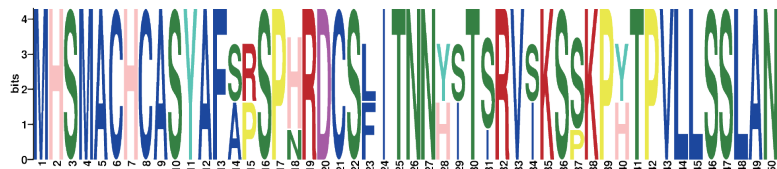

Motif 5

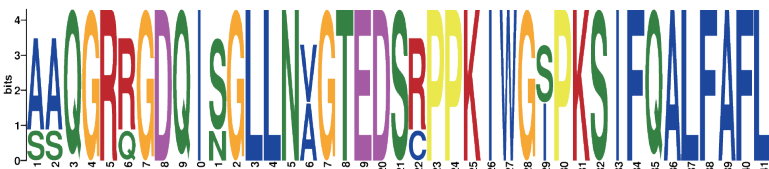

Motif 6

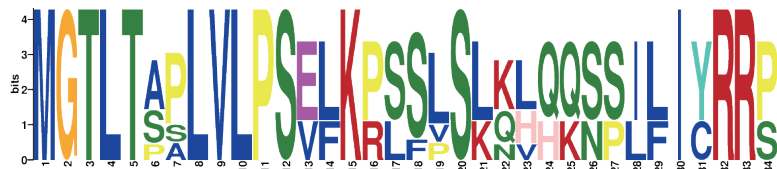

Motif 7

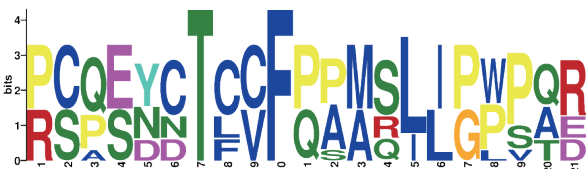

Motif 8

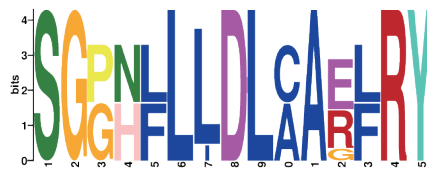

Motif 9

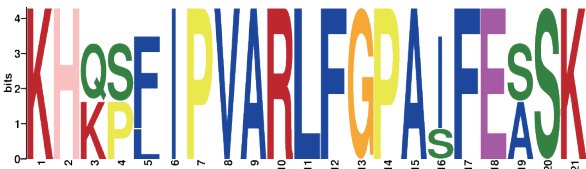

Motif 10

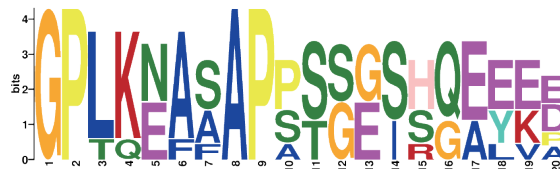

Motif 11

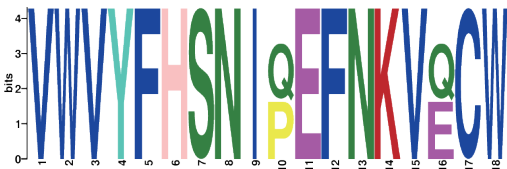

Motif 12

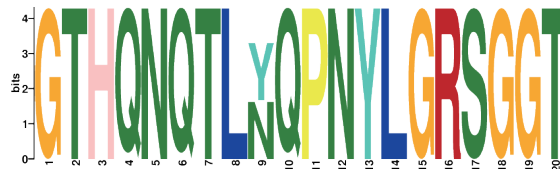

Supplement: Supplementary file 1 [file ijms-24-01993-s001.zip › Figure S1.pdf]

(A)

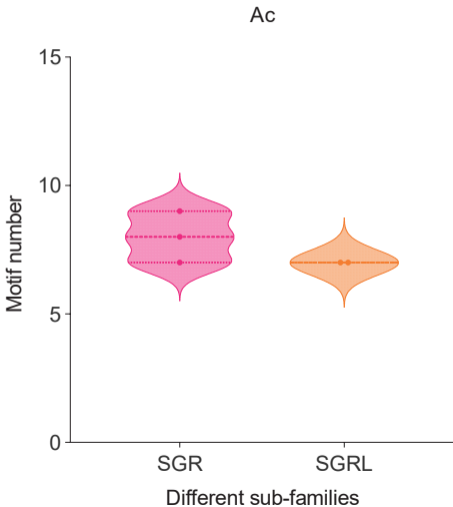

(B)

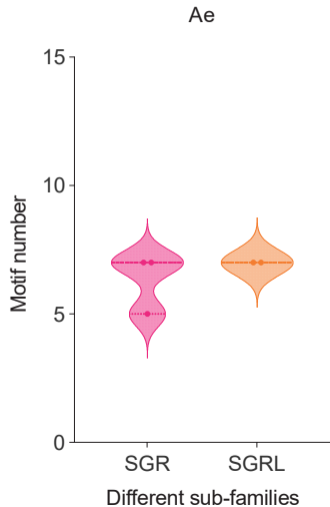

Supplement: Supplementary file 1 [file ijms-24-01993-s001.zip › Figure S2.pdf]

DY

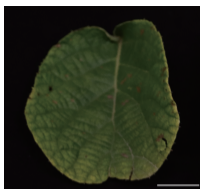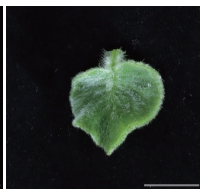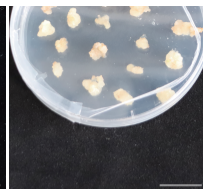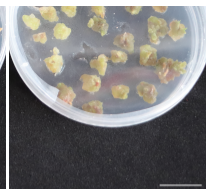

HY

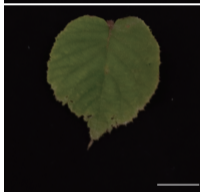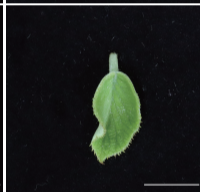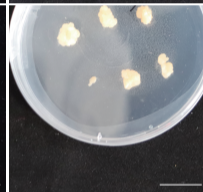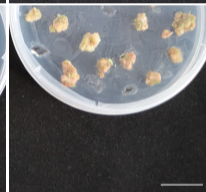

MH

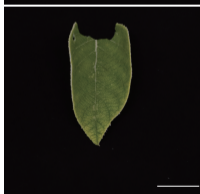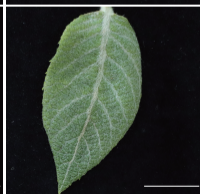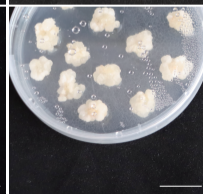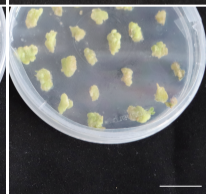

OL

YL

CD

CL

Supplement: Supplementary file 1 [file ijms-24-01993-s001.zip › Figure S3.pdf]

(A)

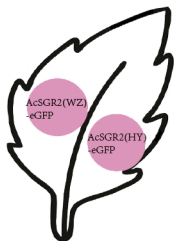

(B)

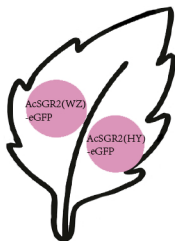

(C)

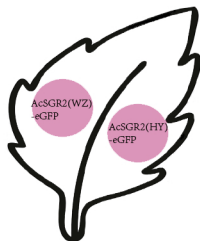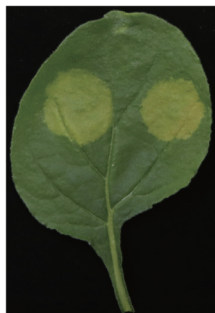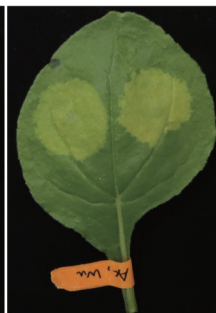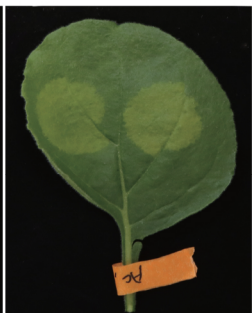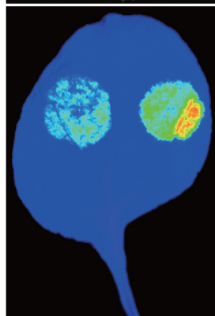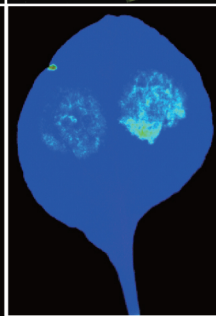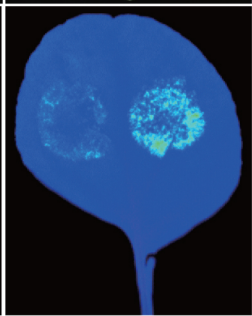

Supplement: Supplementary file 1 [file ijms-24-01993-s001.zip › Figure S5.pdf]

(A)

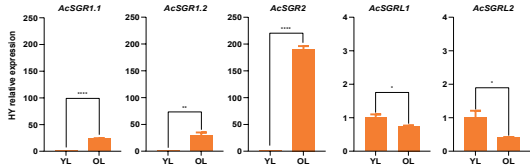

(D)

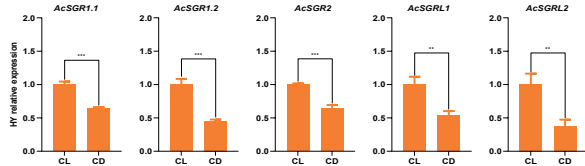

(B)

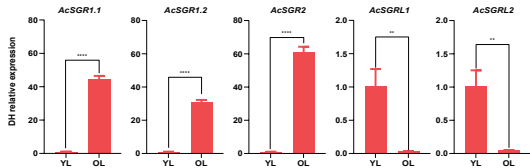

(E)

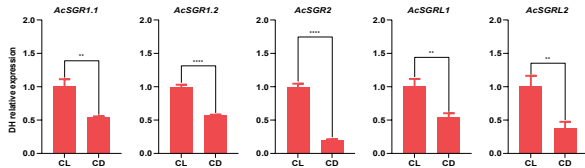

(C)

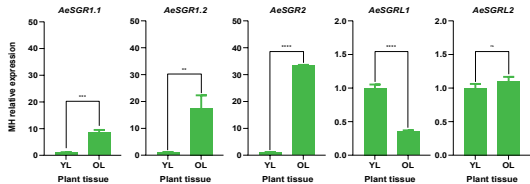

(F)

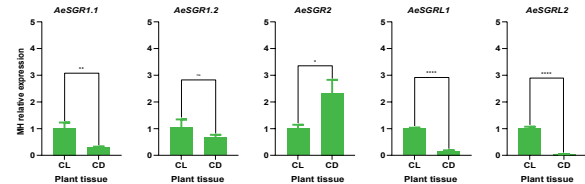

Supplement: Supplementary file 1 [file ijms-24-01993-s001.zip › Figure S6.pdf]
